# Supplementary material for: Introducing non-enzymatic crosslinks into atomistic simulations of collagen fibrils
Source: Bioinformatics. 2026 Jun 23;42(7):btag409. doi: 10.1093/bioinformatics/btag409 (PMC13348703; doi:10.1093/bioinformatics/btag409)
Supplement: btag409_Supplementary_Data [file btag409_supplementary_data.pdf]

Introducing non-enzymatic crosslinks into atomistic simulations of collagen fibrils

Guido Giannetti[1,*], Justin Pils [1], Frauke Gräter [2], Debora Monego[2], Christoph Dellago [1,*],

1 University of Vienna, Faculty of Physics, 1090 Vienna, Austria
2 Max Planck Institute for Polymer Research, Ackermannweg 10, Mainz, Germany

*** Corresponding authors: christoph.dellago@univie.ac.at, guido.giannetti@univie.ac.at**

1 Crosslinks

ColBuilder encodes each intermolecular crosslink in a unified format by specifying the modified residues involved and the corresponding atom–atom connection points. These connection points are highlighted in cyan in this document and are reported as *special bonds* using the standard GROMACS nomenclature. Enzymatic crosslinks typically involve Lys-derived marker residues and are represented by two (divalent) or three (trivalent) connection points. AGE-derived crosslinks involve either Lys–Arg or Lys–Lys residue pairs, are represented by two connection points, and can be specified at sites distributed along the collagen sequence rather than being restricted to the N- and C-terminal regions.

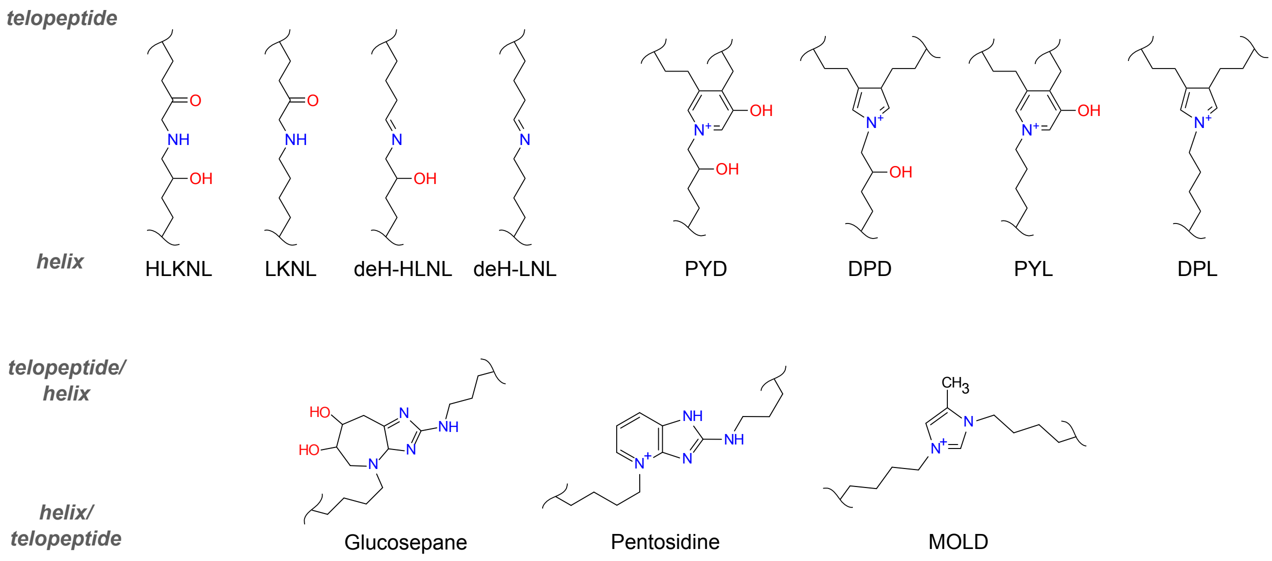

Figure S1. The first row shows the enzymatic crosslink already implemented in [1], which forms at well-defined intermolecular interfaces by coupling a residue in the telopeptide of one collagen molecule to a residue in the triple-helical domain of an adjacent molecule (schematized as telopeptide/helix). The second row shows the three non-enzymatic AGE crosslinks introduced in this work—glucosepane and pentosidine (Lys–Arg-derived) and MOLD (Lys–Lys-derived). These are not restricted to specific collagen regions (schematized as telopeptide/helix or helix/telopeptide), reflecting the fact that AGE formation can in principle involve residues distributed along the full collagen sequence.

2 Crosslinks database (`crosslinks.csv`)

OR	term	combination	type	R1	A1	P1	R2	A2	P2	R3	A31	A32	P3	*#shift*
ARG_LYS	N	310.C – 542.B	Glucosepane	AGS	NZ	310.C	LGX	CE	542.B	NONE	NONE	NONE	NONE	#D3–D4
ARG_LYS	N	509.B - 280.A	Pentosidine	APD	NZ	509.B	LPS	CE	280.A	NONE	NONE	NONE	NONE	#D2–D3
LYS	N	597.A - 358.A	MOLD	LZS	NZ1	597.A	LZD	CE	358.A	NONE	NONE	NONE	NONE	#D3–D4

Table S1. Example of the three AGE-derived crosslink entries in ColBuilder for *Rattus norvegicus*, the template sequence. Columns report the original residue pair (OR), the terminal label (term; required by ColBuilder even for non-terminal AGE sites), the residue pair used for placement (combination), the crosslink chemistry (type), and the residue/atom/position triplets defining the reactive connection points (R1/A1/P1 and R2/A2/P2). The third triplet (R3/A31/A32/P3) is used only for trivalent crosslinks and is set to `NONE` for divalent and AGE-derived crosslinks. Residue positions are given as *residue.chain*. The last column records the required unit cell translation.

3 Number of AGE-derived crosslinks

Species	# Lys_Arg total	# Lys_Arg retained	# Lys_Lys total	# Lys_Lys retained
ailuropoda melanoleuca	88	58	43	24
bos taurus	104	64	41	24
callithrix jacchus	98	63	39	20
canis lupus	104	65	40	20
danio rerio	107	63	36	18
homo sapiens	101	66	39	20
loxodonta africana	95	60	43	21
mus musculus	101	61	42	25
mustela putorius	101	67	39	20
myotis lucifugus	105	67	41	23
oreochromis niloticus	106	65	36	19
oryzias latipes	106	65	36	19
otolemur garnettii	102	65	41	24
pan troglodytes	103	65	41	25
pelodiscus sinensis	97	66	40	16
pongo abelii	98	63	42	23
rattus norvegicus	102	64	45	27
tetraodon nigroviridis	77	47	42	27
xiphophorus maculatus	96	54	41	26

Table S2. Counts of candidate and retained AGE-derived crosslink sites identified across 19 vertebrate collagen type I sequences. Lys–Arg pairs correspond to candidate sites for glucosepane and pentosidine, whereas Lys–Lys pairs correspond to candidate sites for MOLD. "Total" reports the number of residue pairs passing the geometric pre-screening ($C\alpha$–$C\alpha \leq 15$ Å) in the periodic fibril representation, and "retained" reports the subset of candidates that, after explicit model building and local refinement within the fibril environment, satisfy the distance criterion (5 Å) between the reactive atoms forming the crosslink. All species names available in ColBuilder are listed in the first column, and the corresponding crosslink counts (total candidates and retained sites) are reported for each species in the remaining columns.

4 Residue topology and atom charges (*aminoacids.rtp*)

4.1 Glucosepane crosslink

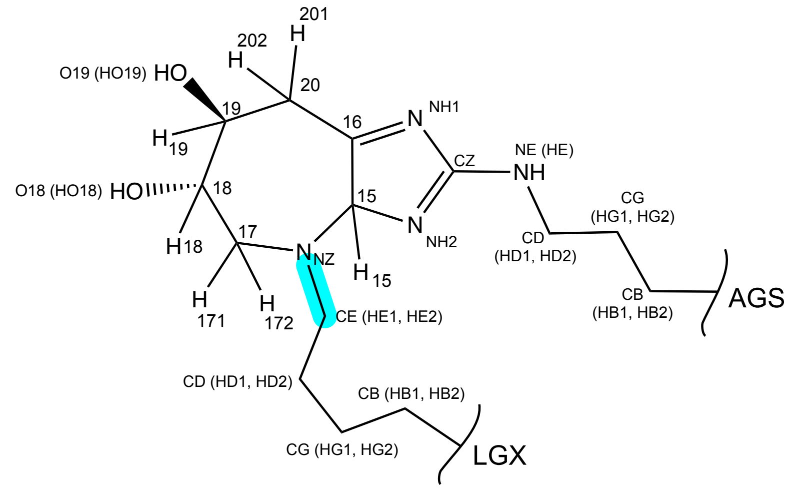

Figure S2. Glucosepane crosslink. Atom names and types as defined in the *aminoacids.rtp* file (in *GROMACS* format). The excerpt below shows the entries corresponding to Glucosepane (`LGX`: modified Lys residue & `AGS`: modified Arg residue).

```
[LGX]
```

[atoms]

N	N	-0.448402	1
H	H	0.244307	2
CA	CT	0.219872	3
HA	H1	0.060908	4
CB	CT	-0.517274	5
HB1	HC	0.160769	6
HB2	HC	0.160769	7
CG	CT	-0.046119	8
HG1	HC	0.028325	9
HG2	HC	0.028325	10
CD	CT	0.069291	11
HD1	HC	0.031814	12
HD2	HC	0.031814	13
CE	CT	-0.453323	14
HE1	H1	0.168580	15
HE2	H1	0.168580	16
C	C	0.587484	18
O	O	-0.561561	19

[bonds]

N	H
N	CA
CA	HA
CA	CB
CA	C
CB	HB1
CB	HB2
CB	CG
CG	HG1
CG	HG2
CG	CD
CD	HD1
CD	HD2
CD	CE
CE	HE1
CE	HE2
C	O
-C	N

[impropers]

-C	CA	N	H
CA	+N	C	O

[AGS]

[atoms]

N	N	-0.409776	1
H	H	0.227143	2
CA	CT	0.134081	3
HA	H1	0.036462	4
CB	CT	-0.030064	5
HB1	HC	0.026185	6
HB2	HC	0.026185	7
CG	CT	0.024089	8
HG1	HC	-0.005728	9
HG2	HC	-0.005728	10
CD	CT	0.122941	11
HD1	H1	0.040543	12
HD2	H1	0.040543	13
NE	N2	-0.649385	14
HE	H	0.368975	15
CZ	C	0.791168	16
NH1	N*	-0.471221	17
NH2	N*	-0.897004	18
C	C	0.506324	19
O	O	-0.523842	20
C15	CT	0.707421	20
H15	H2	-0.022832	21
C16	C	0.019125	22
NZ	N3	-0.085682	17
C17	CT	-0.273185	23
H171	H1	0.111278	24
H172	H1	0.111278	25
C18	CT	0.212995	26
H18	H1	0.106158	27
O18	OH	-0.643348	28
HO18	HO	0.395352	29
C19	CT	0.207711	30
H19	H1	0.003185	31
O19	OH	-0.614835	32
HO19	HO	0.388586	33
C20	CT	-0.069824	34
H201	HC	0.080283	35
H202	HC	0.080283	36

[bonds]

N	H
N	CA
CA	HA
CA	CB
CA	C
CB	HB1
CB	HB2
CB	CG
CG	HG1
CG	HG2
CG	CD
CD	HD1
CD	HD2
CD	NE
NE	HE
NE	CZ
CZ	NH1
CZ	NH2
NH2	C15
NH1	C16
C15	H15
C15	NZ
C17	NZ
C17	H171
C17	H172
C17	C18
C18	H18
C18	O18
O18	HO18
C18	C19
C19	H19
C19	O19
O19	HO19
C20	C19
C20	H201
C20	H202
C20	C16
C16	C15
C	O
-C	N

[impropers]

-C	CA	N	H
CA	+N	C	O
CZ	NH1	NE	NH2
C15	NH2	C16	NZ
C16	C15	NH1	C20

Table S3. Atom names, atom types, atom charges, bonds, and impropers for the `LGX` and `AGS` residues of glucosepane crosslink in *GROMACS* `aminoacids.rtp` format and added to the Amber force field alongside the pre-existing entries [1, 2].

4.1.1 Special Bond *(specialbond.dat)*

resA	atomA	nbondsA	resB	atomB	nbondsB	length	resA	resB
AGS	NZ	1	LGX	CE	1	0.147	AGS	LGX

Table S4. Special bond definition for the glucosepane crosslink between `LGX-CE` and `AGS-NZ`, reported in *GROMACS* `specbond.dat` format and added to the Amber force field alongside the pre-existing entries [1, 2].

4.2 Pentosidine crosslink

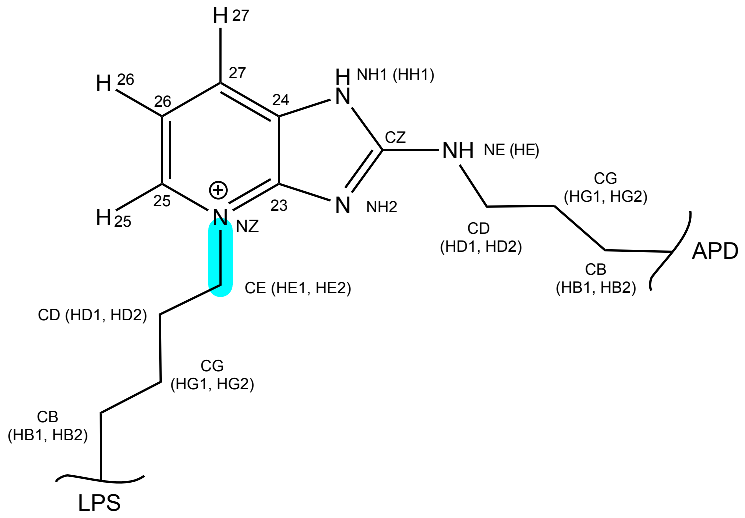

Figure S3. Pentosidine crosslink. Atom names and types as defined in the *aminoacids.rtp* file (in *GROMACS* format). The excerpt below shows the entries corresponding to Pentosidine (`LPS`: modified Lys residue & `APD`: modified Arg residue).

[LPS]

[atoms]

N	N	-0.210936	1
H	H	0.194442	2
CA	CT	-0.135864	3
HA	H1	0.106313	4
CB	CT	-0.129470	5
HB1	HC	0.063701	6
HB2	HC	0.063701	7
CG	CT	0.028893	8
HG1	HC	0.020343	9
HG2	HC	0.020343	10
CD	CT	0.016159	11
HD1	HC	0.029951	12
HD2	HC	0.029951	13
CE	CT	-0.057043	14
HE1	H1	0.093913	15
HE2	H1	0.093913	16
C	C	0.615994	17
O	O	-0.594629	18

[bonds]

N	H
N	CA
CA	HA
CA	CB
CA	C
CB	HB1
CB	HB2
CB	CG
CG	HG1
CG	HG2
CG	CD
CD	HD1
CD	HD2
CD	CE
CE	HE1
CE	HE2
C	O
-C	N

[impropers]

-C	CA	N	H
CA	+N	C	O

[APD]

[atoms]

N	N	-0.295591	1
H	H	0.255825	2
CA	CT	-0.347378	3
HA	H1	0.186036	4
CB	CT	-0.161499	5
HB1	HC	0.071742	6
HB2	HC	0.071742	7
CG	CT	0.162532	8
HG1	HC	0.024357	9
HG2	HC	0.024357	10
CD	CT	-0.075356	11
HD1	H1	0.095229	12
HD2	H1	0.095229	13
NE	N2	-0.555517	14
HE	H	0.328610	15
CZ	CQ	0.887151	16
NH1	NA	-0.796976	17
HH1	H	0.481368	18
NH2	NB	-0.699312	19
C23	CM	0.556462	20
C24	CB	0.187765	21
NZ	N*	-0.077145	22
C25	CM	-0.094801	23
H25	H4	0.202281	24
C26	CA	-0.248796	25
H26	HA	0.214500	26
C27	CA	-0.129208	27
H27	HA	0.220347	28
C	C	0.737116	29
O	O	-0.570742	30

[bonds]

N	H
N	CA
CA	HA
CA	CB
CA	C
CB	HB1
CB	HB2
CB	CG
CG	HG1
CG	HG2
CG	CD
CD	HD1
CD	HD2
CD	NE
NE	HE
NE	CZ
CZ	NH1
CZ	NH2
NH1	HH1
NH1	C24
NH2	C23
C23	C24
C23	NZ
NZ	C25
C25	H25
C25	C26
C26	H26
C26	C27
C27	H27
C27	C24
C	O
-C	N

[impropers]

-C	CA	N	H
CA	+N	C	O
CZ	NH1	NE	NH2
C23	NH2	C24	NZ
C24	C23	NH1	C27

Table S5. Atom names, atom types, atom charges, bonds, and impropers for the `LPS` and `APD` residues of pentosidine crosslink in *GROMACS* `aminoacids.rtp` format and added to the Amber force field alongside the pre-existing entries [1, 2].

4.2.1 Special Bond *(specialbond.dat)*

resA	atomA	nbondsA	resB	atomB	nbondsB	length	resA	resB
LPS	CE	1	APD	NZ	1	0.146	LPS	APD

Table S6. Special bond definition for the pentosidine crosslink between `LPS-CE` and `APD-NZ`, reported in *GROMACS* `specbond.dat` format and added to the Amber force field alongside the pre-existing entries [1, 2].

4.3 MOLD crosslink

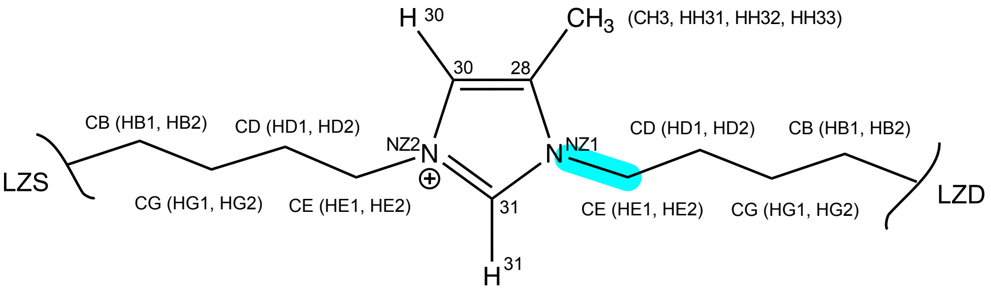

Figure S4. MOLD crosslink. Atom names and types as defined in the *aminoacids.rtp* file (in *GROMACS* format). The excerpt below shows the entries corresponding to MOLD (`LZD`: modified Lys & `LZS`: modified Lys).

`[LZD]`

`[ atoms ]`

N	N	-0.333459	1
H	H	0.244989	2
CA	CT	-0.318983	3
HA	H1	0.163519	4
CB	CT	-0.155066	5
HB1	HC	0.070095	6
HB2	HC	0.070095	7
CG	CT	0.083988	8
HG1	HC	0.059255	9
HG2	HC	0.059255	10
CD	CT	-0.171715	11
HD1	HC	0.081180	12
HD2	HC	0.081180	13
CE	CT	-0.042829	14
HE1	H1	0.100770	15
HE2	H1	0.100770	16
C	C	0.702386	17
O	O	-0.486187	18

`[ bonds ]`

N	H
N	CA
CA	HA
CA	CB
CA	C
CB	HB1
CB	HB2
CB	CG
CG	HG1
CG	HG2
CG	CD
CD	HD1
CD	HD2
CD	CE
CE	HE1
CE	HE2
C	O
-C	N

`[ impropers ]`

-C	CA	N	H
CA	+N	C	O

[LZS]

```
[ atoms ]

   N    N       -0.248165   1
   H    H        0.192753   2
  CA   CT       -0.131209   3
  HA   H1        0.152190   4
  CB   CT       -0.317682   5
 HB1   HC        0.119936   6
 HB2   HC        0.119936   7
  CG   CT       -0.121262   8
 HG1   HC        0.059129   9
 HG2   HC        0.059129  10
  CD   CT        0.179907  11
 HD1   HC        0.006067  12
 HD2   HC        0.006067  13
  CE   CT       -0.211763  14
 HE1   H1        0.132006  15
 HE2   H1        0.132006  16
 NZ2   N*        0.142816  17
   C    C        0.638251  18
   O    O       -0.526574  19
 C31   CQ       -0.043104  20
 H31   H5        0.184965  21
 NZ1   N*       -0.066795  22
 C30   CC       -0.292230  23
 H30   H4        0.231915  24
 C28   CC        0.240186  25
 CH3   CT       -0.412773  26
HH31   HC        0.155018  27
HH32   HC        0.155018  28
HH33   HC        0.155018  29
```

```
[ bonds ]

   N     H
   N    CA
  CA    HA
  CA    CB
  CA     C
  CB   HB1
  CB   HB2
  CB    CG
  CG   HG1
  CG   HG2
  CG    CD
  CD   HD1
  CD   HD2
  CD    CE
  CE   HE1
  CE   HE2
  CE   NZ2
   C     O
  -C     N
 NZ2   C31
 C31   H31
 C31   NZ1
 NZ2   C30
 C30   H30
 C30   C28
 C28   CH3
 CH3  HH31
 CH3  HH32
 CH3  HH33
 C28   NZ1
```

```
[ impropers ]

-C    CA    N     H
CA    +N    C     O
NZ2   C30   CE    C31
C28   NZ1   CH3   C30
```

Table S7. Atom names, atom types, atom charges, bonds, and impropers for the `LZD` and `LZS` residues of MOLD crosslink in *GROMACS* `aminoacids.rtp` format and added to the Amber force field alongside the pre-existing entries [1, 2].

4.3.1 Special Bond *(specialbond.dat)*

resA	atomA	nbondsA	resB	atomB	nbondsB	length	resA	resB
LZS	NZ1	1	LZD	CE	1	0.146	LZS	LZD

Table S8. Special bond definition for the MOLD crosslink between `LZD-CE` and `LZS-NZ1`, reported in *GROMACS* `specbond.dat` format and added to the Amber force field alongside the pre-existing entries [1, 2].

5 Newly introduced bonded parameters for AGEs (*ffbonded.itp*)

Bonds	Atom types	b_0 (nm)	k_b (kJ mol^{-1} nm^{-2})	Crosslink
NE–CZ	N2–C	0.13901	345010.0	Glucosepane (AGS)
NE–CZ	N2–CQ	0.13735	364180.0	Pentosidine (APD)
CZ–NH1	CQ–NA	0.13802	356310.0	Pentosidine (APD)
CZ–NH2	CQ–NB	0.13694	369110.0	Pentosidine (APD)
NH1–C24	NA–CB	0.13802	356310.0	Pentosidine (APD)
NH2–C23	NB–CM	0.13172	439650.0	Pentosidine (APD)
C23–C24	CM–CB	0.14278	351290.0	Pentosidine (APD)
NZ2–C31	N*–CQ	0.13710	367190.0	MOLD (LZS)
NZ2–C30	N*–CC	0.13710	367190.0	MOLD (LZS)
C30–H30	CC–H4	0.10830	292960.0	MOLD (LZS)
**Angles**	**Atom types**	θ_0 (deg)	k_θ (kJ mol^{-1} rad^{-2})	**Crosslink**
CD–NE–CZ	CT–N2–C	120.120	529.690	Glucosepane (AGS)
HE–NE–CZ	H–N2–C	115.680	405.010	Glucosepane (AGS)
NE–CZ–NH1	N2–C–N*	113.640	604.170	Glucosepane (AGS)
NH1–CZ–NH2	N*–C–N*	120.690	606.680	Glucosepane (AGS)
CZ–NH1–C16	C–N*–C	116.010	571.530	Glucosepane (AGS)
NH2–C15–NZ	N*–CT–N3	120.710	561.490	Glucosepane (AGS)
NH2–C15–C16	N*–CT–C	120.710	561.490	Glucosepane (AGS)
H15–C15–C16	H2–CT–C	111.690	390.790	Glucosepane (AGS)
H15–C15–NZ	H2–CT–N3	109.350	415.050	Glucosepane (AGS)
NH1–C16–C15	N*–C–CT	108.720	564.000	Glucosepane (AGS)
CD–NE–CZ	CT–N2–CQ	119.720	533.040	Pentosidine (APD)
HE–NE–CZ	H–N2–CQ	115.630	409.200	Pentosidine (APD)
NE–CZ–NH1	N2–CQ–NA	117.280	600.820	Pentosidine (APD)
NE–CZ–NH2	N2–CQ–NB	117.230	603.330	Pentosidine (APD)
NH1–CZ–NH2	NA–CQ–NB	121.950	590.780	Pentosidine (APD)
CZ–NH1–HH1	CQ–NA–H	125.500	391.620	Pentosidine (APD)
CZ–NH1–C24	CQ–NA–CB	128.010	531.370	Pentosidine (APD)
HH1-NH1–C24	H-NA-CB	125.500	391.620	Pentosidine (APD)
CZ–NH2–C23	CQ–NB–CM	105.490	600.820	Pentosidine (APD)
NH2–C23–C24	NB–CM–CB	112.560	599.150	Pentosidine (APD)
NH2–C23–NZ	NB–CM-N*	112.220	626.760	Pentosidine (APD)
C24–C23–NZ	CB–CM–N*	117.770	574.040	Pentosidine (APD)
NH1–C24-C23	NA–CB–CM	117.770	574.040	Pentosidine (APD)
NH1–C24–C27	NA-CB-CA	106.990	614.210	Pentosidine (APD)
C23–C24–C27	CM–CB–CA	114.190	570.700	Pentosidine (APD)
C25–C26–H26	CM–CA–HA	121.760	405.850	Pentosidine (APD)
CE–NZ2–C31	CT–N*–CQ	125.090	523.500	MOLD (LZS,LZD)
CE–NZ2–C30	CT–N*–CC	125.090	523.500	MOLD (LZS,LZD)
C31–NZ2–C30	CQ–N*–CC	128.010	534.550	MOLD (LZS)
NZ2–C31–H31	N*–CQ–H5	122.100	416.390	MOLD (LZS)
NZ2–C31–NZ1	N*–CQ–N*	129.110	394.890	MOLD (LZS)
NZ2–C30–H30	N*–CC–H4	119.660	420.240	MOLD (LZS)
NZ2–C30–C28	N*–CC–CC	109.420	610.110	MOLD (LZS,LZD)
H30–C30–C28	H4–CC–CC	122.780	548.100	MOLD (LZS)
NZ1–C28–CH3	N*–CC–CT	129.110	394.890	MOLD (LZS)

**Proper torsions**	**Atom types**	ϕ_s (deg)	$\boldsymbol{k_\phi}$ (kJ mol^{-1})	n	**Crosslink**
CD–NE–CZ–NH1	CT–N2–C–N*	180.00	439.320	2	Glucosepane (AGS)
HE–NE–CZ–NH2	H–N2–C–N*	180.00	439.320	2	Glucosepane (AGS)
CD–NE–CZ–NH1	CT–N2–CQ–NA	180.00	4.39320	2	Pentosidine (APD)
CD–NE–CZ–NH2	CT–N2–CQ–NB	180.00	4.39320	2	Pentosidine (APD)
HE–NE–CZ–NH1	H–N2–CQ–NA	180.00	4.39320	2	Pentosidine (APD)
HE–NE–CZ–NH2	H–N2–CQ–NB	180.00	4.39320	2	Pentosidine (APD)
NE–CZ–NH1–HH1	N2–CQ–NA–H	180.00	7.11280	2	Pentosidine (APD)
NE–CZ–NH1–C24	N2–CQ–NA–CB	180.00	7.11280	2	Pentosidine (APD)
NH2–CZ–NH1–HH1	NB–CQ–NA–H	180.00	7.11280	2	Pentosidine (APD)
NH2–CZ–NH1–C24	NB–CQ–NA–CB	180.00	7.11280	2	Pentosidine (APD)
NE–CZ–NH2–C23	N2–CQ–NB–CM	180.00	19.87400	2	Pentosidine (APD)
NH1–CZ–NH2–C23	NA–CQ–NB–CM	180.00	19.87400	2	Pentosidine (APD)
NH2–C23–C24–NH1	NB–CM–CB–NA	180.00	16.73600	2	Pentosidine (APD)
CZ–NH1–C24–C23	CQ–NA–CB–CM	180.00	7.11280	2	Pentosidine (APD)
CZ–NH1–C24–C27	CQ–NA–CB–CA	180.00	7.11280	2	Pentosidine (APD)
HH1—NH1–C24–C23	H–NA–CB–CM	180.00	7.11280	2	Pentosidine (APD)
HH1-NH1–C24–C27	H–NA–CB–CA	180.00	7.11280	2	Pentosidine (APD)
CZ–NH2–C23–C24	CQ–NB–CM–CB	180.00	19.87400	2	Pentosidine (APD)
CZ–NH2–C23–NZ	CQ–NB–CM–N*	180.00	19.87400	2	Pentosidine (APD)
NH2–C23–C24–C27	NB–CM–CB–CA	180.00	16.73600	2	Pentosidine (APD)
NZ–C23–C24–C27	N*–CM–CB–CA	180.00	16.73600	2	Pentosidine (APD)
NZ–C23–C24–NH1	N*–CM–CB–NA	180.00	16.73600	2	Pentosidine (APD)
CE–NZ2–C31–H31	CT–N*–CQ–H5	180.00	7.11280	2	MOLD (LZS,LZD)
CE–NZ2–C31–NZ1	CT–N*–CQ–N*	180.00	7.11280	2	MOLD (LZS,LZD)
C30–NZ2–C31–H31	CC–N*–CQ–H5	180.00	7.11280	2	MOLD (LZS)
C30–NZ2–C31–NZ1	CC–N*–CQ–N*	180.00	7.11280	2	MOLD (LZS)
CE–NZ2–C30–H30	CT–N*–CC–H4	180.00	7.11280	2	MOLD (LZS)
CE–NZ2–C30–C28	CT–N*–CC–CC	180.00	7.11280	2	MOLD (LZS,LZD)
C31–NZ2–C30–H30	CQ–N*–CC–H4	180.00	7.11280	2	MOLD (LZS)
C31–NZ2–C30–C28	CQ–N*–CC–CC	180.00	7.11280	2	MOLD (LZS)
NZ2–C31–NZ1–CE	N*–CQ–N*–CT	180.00	7.11280	2	MOLD (LZS,LZD)
NZ2–C31–NZ1–C28	N*–CQ–N*–CC	180.00	7.11280	2	MOLD (LZS)
CE–NZ1–C28–CH3	CT–N*–CC–CT	180.00	7.11280	2	MOLD (LZS,LZD)
C31–NZ1–C28–C30	CQ–N*–CC–CC	180.00	7.11280	2	MOLD (LZS)
C31–NZ1–C28–CH3	CQ–N*–CC–CT	180.00	7.11280	2	MOLD (LZS)
NZ2–C30–C28–NZ1	N*–CC–CC–N*	180.00	7.11280	2	MOLD (LZS)
NZ2–C30–C28-CH3	N*–CC–CC–CT	180.00	16.73600	2	MOLD (LZS)
H30–C30–C28–NZ1	H4–CC–CC–N*	180.00	16.73600	2	MOLD (LZS)
H30–C30–C28–CH3	H4–CC–CC–CT	180.00	16.73600	2	MOLD (LZS)
**Improper torsions**	**Atom types**	ξ_0 (deg)	$\boldsymbol{k_\xi}$ (kJ mol^{-1} rad^{-2})	n	**Crosslink**
C16–C15–NH1–C20	C–CT–N*–CT	180.00	460.240	2	Glucosepane (AGS)
CZ–NH1–NE–NH2	C–N*–N2–N*	180.00	460.240	2	Glucosepane (AGS)
C15–NH2–C16–NZ	CT–N*–C–N3	180.00	460.240	2	Glucosepane (AGS)
CZ–NH1–NE–NH2	CQ–NA–N2–NB	180.00	460.240	2	Pentosidine (APD)
C23–NH2–C24–NZ	CM–NB–CB–N*	180.00	460.240	2	Pentosidine (APD)
C24–C23–NH1–C27	CB–CM–NA–CA	180.00	4.60240	2	Pentosidine (APD)
NZ2–C30–CE–C31	N*–CC–CT–CQ	180.00	460.240	2	MOLD (LZS,LZD)
C28–CH3–NZ1–C30	CC–CT–N*–CC	180.00	460.240	2	MOLD (LZS,LZD)

Table S9. Bonded parameters for the AGE crosslinks, written in *GROMACS* format, were added to the *ffbonded.itp* file alongside those reported in [1, 2].

6 Benchmarking AGEs Crosslink Force Field Parameters

AGE structures obtained after *ab initio* geometry relaxation were solvated in a 6 nm cubic box of TIP3P water [3] and neutralized with NaCl. Following energy minimization, systems were equilibrated for 10 ns in the NVT ensemble with heavy-atom restraints on the crosslink, then for 15 ns in the NPT ensemble without restraints. Temperature (300 K) and pressure (1 bar) were controlled with the velocity-rescale thermostat [4] and the Parrinello–Rahman barostat [5], and electrostatics were treated with PME [6]. For each crosslink, five independent 100-ns replicas were run with each parameter set (1 µs per crosslink; 3 µs total) using GROMACS 2024.4 [7]. Table S3 reports the ring-angle values for each AGE in the QM-optimized reference structure together with the corresponding replica-averaged values from MD simulations carried out with the two parameter sets, as well as the associated uncertainty, reported as the standard deviation (SD) of the angle distribution across trajectory frames. Angle definitions follow the atom nomenclature in Figures S2–S4. Angles involving bonds between ring atoms and side-chain atoms and/or ring substituents (C–OH and C–CH_3 substituents in glucosepane and MOLD, respectively) are highlighted in yellow. Figures S5 presents RMSD, RMSF, and end-to-end distance (E2E) structural metrics evaluated relative to the QM structure.

Glucosepane

Angle (deg)	*QM*	*Antechamber*	*Grappa*	*Angle (deg)*	*QM*	*Antechamber*	*Grappa*
NH1−CZ−NH2	117.25	115.20 ± 2.7	116.28 ± 2.05	C19−C20−C16	108.76	114.43 ± 3.15	111.21 ± 3.62
NH2−CZ−NE	125.10	111.28 ± 4.70	121.38 ± 2.72	C18−C19−C20	115.89	114.17 ± 3.60	114.16 ± 3.23
NH1−CZ−NE	117.63	116.79 ± 4.33	122.03 ± 2.64	C18−C19−O19	109.11	109.42 ± 3.69	109.06 ± 3.14
C16−NH1−CZ	103.89	108.42 ± 2.63	104.98 ± 2.08	C20−C19−O19	107.85	107.12 ± 3.68	109.15 ± 3.10
C15−NH2−CZ	103.88	103.11 ± 2.52	103.82 ± 2.07	C17−C18−C19	117.03	114.88 ± 3.76	114.32 ± 3.31
NH2−C15−C16	103.67	106.99 ± 2.42	104.02 ± 1.97	C17−C18−O18	111.47	105.06 ± 3.77	108.49 ± 3.21
NZ−C15−NH2	117.84	118.78 ± 3.24	115.96 ± 3.40	C19−C18−O18	107.71	110.13 ± 3.66	108.82 ± 3.15
NZ−C15−C16	112.93	111.75 ± 2.92	112.80 ± 3.31	NZ−C17−C18	111.40	112.63 ± 3.22	115.12 ± 3.90
C15−C16−NH1	110.82	104.27 ± 2.52	110.40 ± 2.03	C17−NZ−CE	118.19	112.10 ± 3.84	117.29 ± 3.62
C20−C16−C15	124.0	124.61 ± 3.19	123.24 ± 3.11	C15−NZ−CE	114.47	116.10 ± 3.96	114.76 ± 3.58

Pentosidine

Angle (deg)	*QM*	*Antechamber*	*Grappa*	*Angle (deg)*	*QM*	*Antechamber*	*Grappa*
NH1−CZ−NH2	112.68	116.74 ± 2.28	112.61 ± 1.99	C23−C24−C27	121.63	122.77 ± 2.27	122.55 ± 2.42
NE−CZ−NH1	122.84	121.94 ± 3.17	120.55 ± 2.83	C24−NH1−CZ	107.32	101.52 ± 2.20	106.53 ± 1.97
NH2−CZ−NE	124.46	119.97 ± 3.26	126.49 ± 2.69	C24−C27−C26	116.63	115.26 ± 2.28	114.74 ± 2.45
CZ−NH2−C23	104.99	100.79 ± 2.14	104.74 ± 1.99	C27−C26−C25	119.79	120.86 ± 2.27	122.02 ± 2.47
NH2−C23−C24	112.15	110.59 ± 2.11	112.54 ± 1.99	C26−C25−NZ	122.49	120.73 ± 2.22	121.31 ± 2.37
C25−NZ−C23	118.86	117.14 ± 2.18	118.73 ± 2.37	C25−NZ−CE	120.88	120.26 ± 2.62	121.43 ± 2.82
C24−C23−NZ	120.56	122.03 ± 2.18	119.70 ± 2.37	C23−NZ−CE	120.25	121.96 ± 2.59	119.27 ± 2.84
C23−C24−NH1	102.82	109.74 ± 2.17	103.12 ± 1.957				

MOLD

Angle (deg)	*QM*	*Antechamber*	*Grappa*	*Angle (deg)*	*QM*	*Antechamber*	*Grappa*
NZ2−C31−NZ1	109.35	106.77 ± 2.34	108.17 ± 2.01	C30−C28−CH3	129.43	121.48 ± 2.85	129.28 ± 3.18
C31−NZ1−C28	108.5	109.79 ± 2.25	109.29 ± 2.01	C28−C30−NZ2	108.62	104.51 ± 2.11	107.16 ± 2.03
C31−NZ1−CE	123.18	124.08 ± 2.88	123.26 ± 3.17	C30−NZ2−C31	107.65	111.40 ± 2.27	108.89 ± 2.05
CE−NZ1−C28	128.29	125.43 ± 2.84	126.79 ± 3.12	C30−NZ2−CE	124.68	123.74 ± 2.96	125.39 ± 3.28
NZ1−C28−C30	105.86	106.81 ± 2.11	106.13 ± 2.01	C31−NZ2−CE	127.65	124.21 ± 2.95	125.22 ± 3.27
NZ1−C28−CH3	124.70	130.65 ± 2.81	124.22 ± 3.07				

Table S10. Ring-angle geometry of AGE crosslinks. QM values are taken from the DFT-optimized reference structures; MD values (Antechamber and Grappa) are reported as mean ± standard deviation over trajectory frames. Angle definitions follow Figures S2–S4. Angles highlighted in yellow involve bonds between ring atoms and side-chain atoms and/or ring substituents and are shown for completeness but were not included in the ring-core benchmarking discussed in the main text.

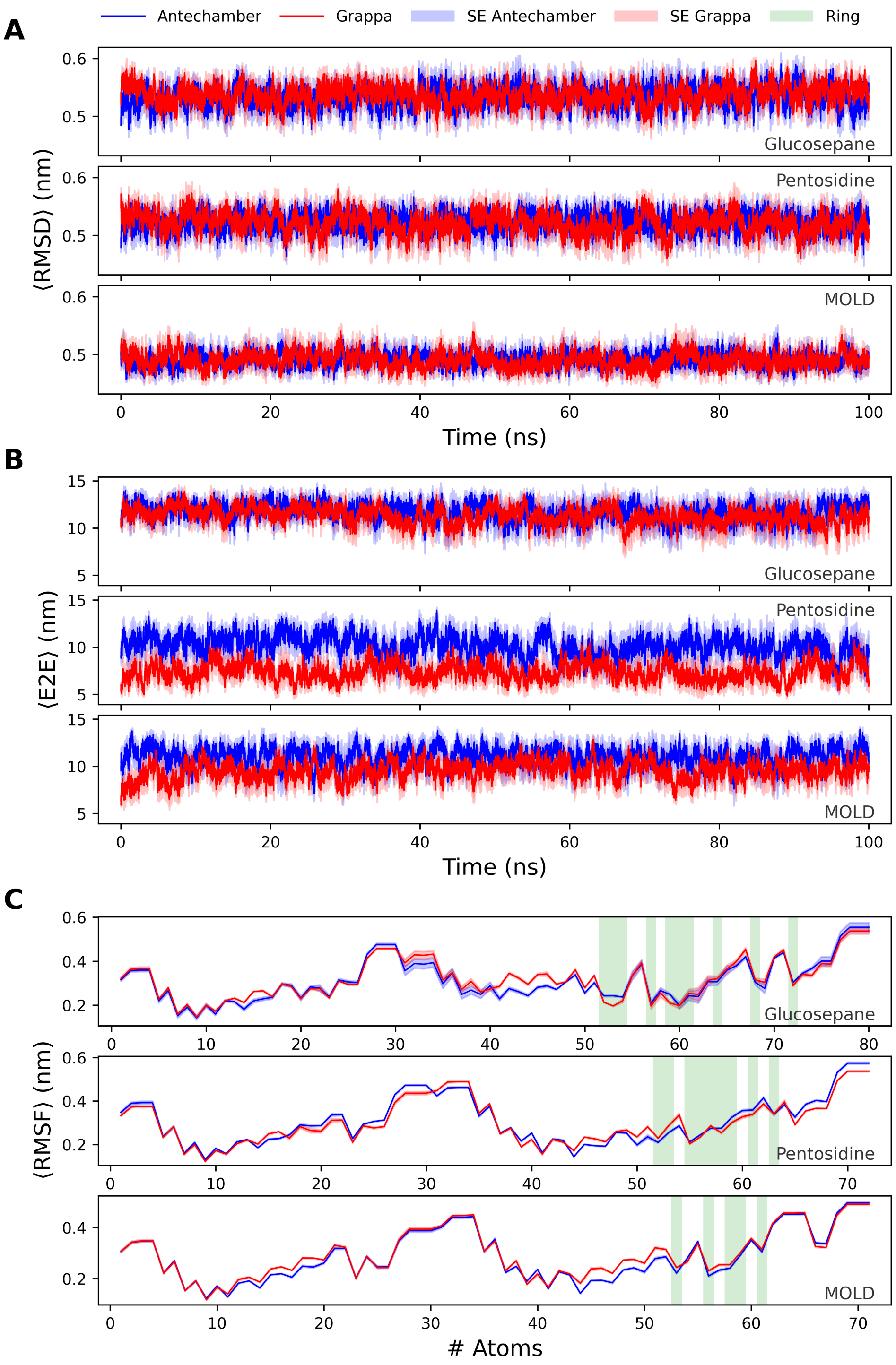

Figure S5. Structural metrics of AGE crosslinks from MD simulations. Blue and red curves correspond to the Antechamber and Grappa parameter sets, respectively; shaded regions indicate the standard error of the mean (SEM) across replicas. **(A)** ⟨RMSD⟩ of the crosslink (all atoms) relative to the QM reference structure as a function of time. **(B)** ⟨E2E⟩, defined as the distance between the two crosslink Cα atoms, as a function of time. **(C)** ⟨RMSF⟩ (all atoms) relative to the QM reference structure; green bands highlight ring atoms. Time-averaged values are reported in Table S11.

Structural metrics	Crosslink type	Antechamber	Grappa
$\langle$RMSD$\rangle$ (nm)	Glucosepane	0.533 ± 0.015	0.537 ± 0.015
	Pentosidine	0.524 ± 0.013	0.521 ± 0.017
	MOLD	0.491 ± 0.010	0.490 ± 0.012
$\langle$RMSD$\rangle_{\text{ring}}$ (nm)	Glucosepane	0.124 ± 0.001	0.126 ± 0.001
	Pentosidine	0.139 ± 0.001	0.135 ± 0.001
	MOLD	0.129 ± 0.001	0.129 ± 0.001
$\langle$E2E$\rangle$ (nm)	Glucosepane	11.700 ± 0.847	11.321 ± 0.853
	Pentosidine	10.154 ± 0.919	7.356 ± 0.824
	MOLD	11.066 ± 0.912	9.523 ± 0.861
$\langle$RMSF$\rangle$ (nm)	Glucosepane	0.310 ± 0.011	0.317 ± 0.008
	Pentosidine	0.308 ± 0.004	0.302 ± 0.004
	MOLD	0.287 ± 0.003	0.297 ± 0.003
$\langle$RMSF$\rangle_{\text{ring}}$ (nm)	Glucosepane	0.254 ± 0.009	0.242 ± 0.009
	Pentosidine	0.277 ± 0.003	0.275 ± 0.005
	MOLD	0.297 ± 0.003	0.269 ± 0.002

Table S11. Time-averaged structural metrics for AGE crosslinks. Values are reported as mean $\pm$ SEM across replicas. Blue/red: Antechamber/Grappa. $\langle$RMSD$\rangle$ (Figure S5A) is computed relative to the QM reference structure; $\langle$RMSD$\rangle_{\text{ring}}$ and $\langle$RMSF$\rangle_{\text{ring}}$ are computed using ring atoms only; $\langle$E2E$\rangle$ (Figure S5B) is the Cα–Cα distance between the two crosslinking residues; $\langle$RMSF$\rangle$ (Figure S5C) quantifies per-atom flexibility around the mean structure.

7 Collagen microfibril all-atom simulations

Geometric observables were computed from *mean positions* (centers of geometry) of predefined atom groups. The end-to-end distance was calculated for each frame as the Euclidean distance between the mean positions of the ACE (N-terminus) and NME (C-terminus) cap residues, and is reported as a proxy for the molecular extension (D-band length).

To partition the extension into overlap and gap contributions, we identified the crosslink sets located at the N- and C-terminal sites and used their positions for per-frame clustering. For the PYD and PYD + glucosepane systems, enzymatic crosslinks were detected from the bond network as connected components restricted to the trivalent marker residues (`LYX`, `LY2`, `LY3`), retaining only valid trios containing one residue of each type. For the AGE-terminated systems, crosslinks were instead represented as marker *pairs* and clustered accordingly: `AGS`/`LGX` for glucosepane, `LPS`/`APD` for pentosidine, and `LZS`/`LZD` for MOLD. In all cases, a crosslink position in a given frame was defined as the mean position of all atoms belonging to the corresponding marker set (trio for PYD; pair for AGEs).

For each frame, marker-set mean positions were projected onto the instantaneous molecular axis defined by the ACE$\rightarrow$NME direction. The resulting one-dimensional coordinates were segmented using $k = 2$ K-means clustering, yielding two clusters corresponding to crosslinks located closer to the N-terminus and C-terminus, respectively. The *overlap* length was defined as the axial separation between the two cluster centroids, and the *gap* length as the residual extension (gap = end-to-end − overlap). This two-cluster segmentation is appropriate for the present reduced system length, where only two terminal crosslink groups are resolved along the fibril axis.

Uncertainties for overlap and gap were estimated from the within-cluster spread of projected crosslink coordinates by computing standard errors of the cluster means and propagating them to the overlap (difference of cluster centroids) and gap (end-to-end minus overlap). For the end-to-end distance, we combined (i) the propagated frame-wise contribution arising from overlap and gap uncertainties with (ii) a trajectory-scale bootstrap estimate obtained using a 10-frame sliding window and 1000 resamples per window; the two contributions were combined by a root-mean-square procedure. The overlap/gap strain ratio was computed relative to the first analyzed frame

as $(Overlap(t) - Overlap(t_0))/(Gap(t) - Gap(t_0))$, with uncertainty propagated from the corresponding overlap and gap errors. Three independent replicas with distinct initial velocities were analyzed. Replica-averaged time series were obtained by arithmetic averaging, and uncertainties on the averaged curves were computed by combining per-replica errors in quadrature and scaling by the number of replicas.

Here, we report a detailed summary of all simulated model cases, including the crosslink type, number of crosslinks, and relaxed and pulled lengths of the D-period, overlap, and gap regions:

Property	glucosepane	MOLD	PYD+glucosepane	pentosidine	PYD
Crosslink type(s)	glucosepane	MOLD	PYD + glucosepane	pentosidine	PYD
Crosslink count(s)	20	20	20 + 10	20	20
$D_{t=0}$ (nm)	67.118	67.170	67.213	67.161	67.135
$D_{t=100}$ (nm)	83.257	83.454	83.154	83.234	83.185
$O_{t=0}$ (nm)	27.429	27.674	27.397	27.936	27.419
$O_{t=100}$ (nm)	32.526	32.333	31.552	32.478	31.612
$G_{t=0}$ (nm)	39.689	39.496	39.816	39.224	39.716
$G_{t=100}$ (nm)	50.731	51.119	51.602	50.756	51.572

Table S12. Summary of the model systems used in Fig. 4. Values are reported for the initial frame ($t = 0$ ns) and the final frame ($t = 100$ ns). For the mixed system, crosslink counts are reported as PYD + glucosepane, with glucosepane crosslinks added in the overlap region.

7.1 Estimation of the fibril-level Poisson ratio

To quantify the radial response of the fibrillar segment during pulling, we computed an effective geometrical Poisson ratio from the relaxed and pulled structures. Solvent and ions were excluded from the analysis. The fibril axis was defined as the vector connecting the mean positions of the ACE and NME capping groups. The fibril was divided into 20 material slices along the ACE-to-NME axis in the relaxed structure, each corresponding to 5% of the initial fibril length. Atom indices assigned to each slice in the relaxed structure were then followed in the pulled structure, ensuring that the same material portion of the fibril was compared before and after elongation.

For each slice and for each structure, atomic positions were projected onto the plane perpendicular to the instantaneous ACE-to-NME axis. The local transverse radius was defined as the 95th percentile of the radial distances from the transverse center of the slice, providing a robust estimate of the outer fibrillar radius while avoiding sensitivity to single peripheral atoms. The transverse strain of each slice was computed as

$$\varepsilon_\perp = \frac{R_{95}^{\text{pull}} - R_{95}^{\text{relaxed}}}{R_{95}^{\text{relaxed}}},$$

whereas the axial strain was computed from the ACE-to-NME distance as

$$\varepsilon_\parallel = \frac{L^{\text{pull}} - L^{\text{relaxed}}}{L^{\text{relaxed}}}.$$

The effective Poisson ratio was then calculated as

$$\nu_{\text{eff}} = -\frac{\varepsilon_\perp}{\varepsilon_\parallel}.$$

Values reported in the main text correspond to atom-count-weighted averages over all 20 material slices of each fibril. This analysis yielded ν_{eff} values of 0.65, 0.59, 0.58, 0.62, and 0.66 for the glucosepane, MOLD, pentosidine, PYD-only, and PYD+glucosepane systems, respectively. These values should be interpreted as effective geometrical Poisson ratios of the simulated fibrillar segment rather than as elastic constants derived from deformation of the simulation cell.

8 Extending the ColBuilder framework with new crosslinks

This section provides a practical guide for users who wish to add crosslink chemistries that are not yet included in the ColBuilder database. We outline the minimal set of edits required to (i) register a new crosslink type for model building and geometry refinement and (ii) enable all-atom topology export by providing compatible Amber99 force-field parameters.

Crosslink representation and database entry. In ColBuilder, each crosslink is encoded using custom *marker residues* that represent the chemically modified amino acids participating in bond formation. Divalent crosslinks are represented by two markers, whereas trivalent crosslinks are represented by three. How the underlying chemistry is partitioned across markers is a modeling choice (e.g., assigning a ring system to one marker), but the pipeline requires that marker identities and their forming atoms are defined consistently.

New crosslinks are registered by adding one row per crosslink definition to `colbuilder/src/colbuilder/data/sequence/crosslinks.csv` (see Table S1 for an example). In practice, entries are selected by matching (i) the `term` flag (`N` or `C`), (ii) the `type` label (the crosslink name selected in the configuration), and (iii) the `combination` string (a user-defined key matched exactly). Each row specifies the marker residues and their forming atoms via `R1/A1/P1`, `R2/A2/P2` (and `R3/A31/P3` for trivalent links), where positions `P` are given as `resnum.chain` (e.g., `1004.B`). The `#shift` field is retained as an annotation to record the unit-cell translation required to optimize the crosslink.

MODELLER residue registration. Each marker must be available to MODELLER as a residue type. Concretely, markers are mapped to single-character residue identifiers in `colbuilder/src/colbuilder/data/sequence/modeller/restyp_mod.lib`, and their heavy-atom topology is added to `colbuilder/src/colbuilder/data/sequence/modeller/top_heav_mod.lib`. To streamline the latter step, we provide a helper script in `https://github.com/gggguido/AGEs-ColBuilder.git` that generates a copy/paste-ready topology block for `top_heav_mod.lib` starting from a marker geometry specified as a SMILES string. The script (`create_ic_crosslink.py`) builds a 3D conformer with RDKit, assigns systematic atom naming, and writes an internal-coordinate (IC) specification together with the required bonded connectivity, reducing manual editing when defining additional markers.

Geometry pipeline integration. To ensure that the geometry and refinement routines recognize the new markers, the marker lists and crosslink parsing logic must be extended in `colbuilder/src/colbuilder/core/geometry/crosslink.py` and `colbuilder/src/colbuilder/core/geometry/connect.py`. In addition, the pairing/trio rules and replacement mappings that support crosslink mixing, correction of unpaired markers, and user-controlled marker replacement must be updated in `colbuilder/src/colbuilder/core/geometry/geometry_replacer.py`, `colbuilder/src/colbuilder/core/geometry/unpaired_crosslinks.py`, and `colbuilder/src/colbuilder/core/geometry/crosslink_mixer.py`. Collectively, these files define which marker residues are considered compatible within a crosslink and how markers are reverted to their corresponding standard residues (e.g., Lys/Arg) when required by specific workflow operations.

All-atom parameterization (required for topology export). If the new markers are intended for all-atom molecular dynamics, they must be parameterized and made available to the topology builder. We followed a standard AMBER/GAFF workflow, using AmberTools [8], based on quantum-mechanical geometry optimization and electrostatic-potential (ESP) fitting. For each marker, we ran a Gaussian calculation at the B3LYP/6-31G* level to optimize the geometry and compute a Merz–Kollman ESP (via `Pop=MK` and the associated `iop` settings), yielding the Gaussian output and an ESP grid (`.gesp`). The ESP grid was converted to a `.esp` file using `espgen` and used for a two-stage RESP fit: starting from the Gaussian output, `antechamber` generated an initial `.ac` file, `respgen` prepared `respin1/respin2` (and `qin`), and RESP stage 1 and stage 2 fits produced the final restrained charge set. In the stage 2 fit, charges on the acetyl (`ACE`) and N-methyl (`NME`) caps were constrained to reproduce the corresponding AMBER values by freezing the cap-atom charges through the `qin`/constraint specification used by `respgen`. The resulting RESP charges were assigned back to the structure with `antechamber`; GAFF atom types were then applied, and missing bonded parameters were identified with `parmchk` to generate the `.frcmod`

file. Finally, `.mol2` (RESP charges) and `.prepi/.frcmod` files were produced for LEaP, enabling generation of AMBER topology/coordinate files (`.prmtop/.inpcrd`) for subsequent conversion via `acpype` and use in *GROMACS* simulations. A full example is reported in `https://github.com/gggguido/AGEs-ColBuilder.git`.

All-atom topology generation (Amber99SB*-ildnp). For ColBuilder to export all-atom topologies, the topology layer must recognize each marker residue and its bonding atom. This mapping is defined in `colbuilder/src/colbuilder/core/topology/amber.py`. The force-field data files then provide atom types, partial charges, hydrogen-generation rules, residue classification, and special-bond definitions. For Amber99sb*-ildnp, these edits are made in `colbuilder/src/colbuilder/data/topology/amber99sb-star-ildnp.ff/`, specifically in `aminoacids.rtp` (residue definitions), `specbond.dat` (special crosslink bonds), `residuetypes.dat` (residue classification), and `aminoacids.hdb` (hydrogen construction rules). If Amber99 output is also required, the corresponding residue definition must additionally be added in `colbuilder/src/colbuilder/data/topology/amber99/` (file: `aminoacids.rtp`).

References

[1] Agnieszka Obarska-Kosinska, Benedikt Rennekamp, Aysecan Ünal, and Frauke Gräter. ColBuilder: A server to build collagen fibril models. *Biophys. J.*, 120(17):3544–3549, 2021.

[2] Debora Monego, Matthias Brosz, Johanna Buck, Vsevolod Viliuga, Paulius Greicius, Jaewoon Jung, Torsten Stuehn, Matthias Schmies, Yuji Sugita, and Frauke Gräter. ColBuilder: flexible structure generation of crosslinked collagen fibrils. *Bioinformatics*, 41(6):btaf278, 2025.

[3] William L Jorgensen, Jayaraman Chandrasekhar, Jeffry D Madura, Roger W Impey, and Michael L Klein. Comparison of simple potential functions for simulating liquid water. *J. Chem. Phys.*, 79:926–935, 1983.

[4] Giovanni Bussi, Davide Donadio, and Michele Parrinello. Canonical sampling through velocity rescaling. *J. Chem. Phys.*, 126(1):014101, 2007.

[5] M Parrinello and A Rahman. Polymorphic transitions in single crystals: A new molecular dynamics method. *J. Appl. Phys.*, 52(12):7182–7190, 1981.

[6] Tom Darden, Darrin York, and Lee Pedersen. Particle mesh ewald: an nlog(n) method for ewald sums in large systems. *J. Chem. Phys.*, 98(12):10089–10092, 1993.

[7] Van Der Spoel, David and Lindahl, Erik and Hess, Berk and Groenhof, Gerrit and Mark, Alan E. and Berendsen, Herman J. C. GROMACS: Fast, flexible, and free. *J. Comp. Chem.*, 26(16):1701–1718, 2005.

[8] David A. Case, Hasan Metin Aktulga, Kellon Belfon, David S. Cerutti, G. Andrés Cisneros, Vinícius Wilian D. Cruzeiro, Negin Forouzesh, Timothy J. Giese, Andreas W. Götz, Holger Gohlke, Saeed Izadi, Koushik Kasavajhala, Mehmet C. Kaymak, Edward King, Tom Kurtzman, Tai-Sung Lee, Pengfei Li, Jian Liu, Tyler Luchko, Ray Luo, Madushanka Manathunga, Matias R. Machado, Hai Minh Nguyen, Kurt A. O'Hearn, Alexey V. Onufriev, Feng Pan, Sergio Pantano, Ruxi Qi, Ali Rahnamoun, Ali Risheh, Stephan Schott-Verdugo, Akhil Shajan, Jason Swails, Junmei Wang, Haixin Wei, Xiongwu Wu, Yongxian Wu, Shi Zhang, Shiji Zhao, Qiang Zhu, Thomas E. III Cheatham, Daniel R. Roe, Adrian Roitberg, Carlos Simmerling, Darrin M. York, Maria C. Nagan, and Kenneth M. Jr. Merz. AmberTools. *J. Chem. Inf. Model.*, 63(20):6183–6191, 2023.
